# Retraction: Molecular Evidence for the Presence of *Rickettsia Felis* in the Feces of Wild-living African Apes

**DOI:** 10.1371/journal.pone.0312332

**Published:** 2024-10-14

**Authors:** The PLOS ONE Editors

The *PLOS ONE* Editors retract this article [1, 2] due to concerns about compliance with the PLOS Animal Research policy and *PLOS ONE*’s guidelines for articles reporting observational and field studies.

This study [1, 2] involved collection of samples in Cameroon and Democratic Republic of Congo, from species categorized by IUCN [3] as endangered (chimpanzee, bonobo) and critically endangered (gorilla).

PLOS received a copy of the ethics approval document N°259/CNE/SE/201 cited in the Ethics Statement of [1]. It appears to cover a different study than that reported in this article: N°259/CNE/SE/201 was issued by the Comité National d’Ethique in Cameroon for an SIV study (protocol title: “*Identification et prévalences des SIVs chez les primates non-humains sauvages afin d’estimer les risques de nouvelles transmissions inter-espèces et d’étudier plus en détails les réservoirs des ancêtres due HIV-1 chez les grands singes en Afrique Centrale*”). The document does not mention collections in the Democratic Republic of Congo or investigation of *Rickettsia felis*. (Ethics approval N°259/CNE/SE/201 was also cited in [4], which reports a *Plasmodium falciparum* study in Cameroon.)

In response to PLOS’ queries the authors did not clarify this issue or provide documentation to confirm that the study complied with applicable research regulations in the Democratic Republic of Congo and received local approval for all field collections.

A representative of the Aix-Marseille Université Ethics Committee stated that the institute disagrees with the retraction decision, that the cited approval from Cameroon authorized the research in [1, 2], and that the study complied with the local legislation and international ethics standards. PLOS concluded that the response from Aix-Marseille Université did not resolve the above concerns.

DR indicated that he disagrees with the retraction decision. AKK, CS, SAM, PR, CB, AA, BII, JJMT, EMN, ED, MP, and FF either did not respond directly or could not be reached.
